# Novel genetic mutations detected by multigene panel are associated with hereditary colorectal cancer predisposition

**DOI:** 10.1371/journal.pone.0203885

**Published:** 2018-09-26

**Authors:** Lorena Martin-Morales, Paula Rofes, Eduardo Diaz-Rubio, Patricia Llovet, Victor Lorca, Inmaculada Bando, Pedro Perez-Segura, Miguel de la Hoya, Pilar Garre, Vanesa Garcia-Barberan, Trinidad Caldes

**Affiliations:** 1 Molecular Oncology Laboratory, Hospital Clinico San Carlos, IdISSC, Madrid, Spain; 2 CIBERONC (Centro de Investigacion Biomedica en Red de Cancer), Carlos III Health Institute, Madrid, Spain; 3 Medical Oncology, Hospital Clinico San Carlos, Madrid, Spain; Ohio State University Wexner Medical Center, UNITED STATES

## Abstract

Half of the high-risk colorectal cancer families that fulfill the clinical criteria for Lynch syndrome lack germline mutations in the mismatch repair (MMR) genes and remain unexplained. Genetic testing for hereditary cancers is rapidly evolving due to the introduction of multigene panels, which may identify more mutations than the old screening methods. The aim of this study is the use of a Next Generation Sequencing panel in order to find the genes involved in the cancer predisposition of these families. For this study, 98 patients from these unexplained families were tested with a multigene panel targeting 94 genes involved in cancer predisposition. The mutations found were validated by Sanger sequencing and the segregation was studied when possible. We identified 19 likely pathogenic variants in 18 patients. Out of these, 8 were found in MMR genes (5 in *MLH1*, 1 in *MSH6* and 2 in *PMS2*). In addition, 11 mutations were detected in other genes, including high penetrance genes (*APC*, *SMAD4* and *TP53*) and moderate penetrance genes (*BRIP1*, *CHEK2*, *MUTYH*, *HNF1A* and *XPC*). Mutations c.1194G>A in *SMAD4*, c.714_720dup in *PMS2*, c.2050T>G in *MLH1* and c.1635_1636del in *MSH6* were novel. In conclusion, the detection of new pathogenic mutations in high and moderate penetrance genes could contribute to the explanation of the heritability of colorectal cancer, changing the individual clinical management. Multigene panel testing is a more effective method to identify germline variants in cancer patients compared to single-gene approaches and should be therefore included in clinical laboratories.

## Introduction

Hereditary Non-Polyposis Colorectal Cancer (HNPCC) is a familial syndrome with an increased incidence of colorectal (CRC) and other related cancers [1,2], defined by the Amsterdam I or II clinical criteria. It is well established that approximately half of HNPCC cases are explained by germline mutations in the DNA mismatch repair (MMR) genes, mainly *MLH1*, *MSH2*, *MSH6* and *PMS2*. As a consequence, these cases present MMR pathway defects and microsatellite instability (MSI) in the tumors, and are referred to as Lynch Syndrome (LS) [3,4].

The universal screening for LS currently comprises two different stages. Firstly, the immunohistochemistry (IHC) of the MMR proteins and/or the MSI status is studied in the tumor of every CRC patient, as well as in some endometrial cancers [5,6]. When this result is positive (MSI/absence of MMR) or if the tumor is not available, patients with a family history of CRC are then screened for germline mutations in the MMR genes, which was previously performed by methods such as Denaturation Gradient Gel Electrophoresis (DGGE) or High Resolution Melting (HRM), followed by Sanger sequencing of samples with altered patterns. Initially, candidates for this LS genetic testing were identified based on the Amsterdam criteria [7,8] However, these algorithms may miss some individuals with LS [9], reason for which the more lenient Bethesda guidelines were created to identify high-risk families that should undergo genetic testing. Although all of these are effective screening tools, they may still miss a proportion of patients with LS. It is worth noting that the IHC and MSI tests have lower sensitivity for detecting *MSH6* and *PMS2* mutation carriers in particular [9], and that the screening of *PMS2* is a challenge due to the high number of pseudogenes.

After this screening, only those families in which a germline pathogenic mutation is found in one of the MMR genes are diagnosed with Lynch Syndrome. Those cases that show MSI/MMR defects in the tumor but lack the corresponding germline MMR mutations are classified as unexplained MMR deficiency, whereas the other half of HNPCC families with no evidence of MMR deficiency has been designated Familial Colorectal Cancer Type X (FCCTX) [10–13]. FCCTX patients lack germline MMR mutations and their tumors are microsatellite stable (MSS). The lack of information about the hereditability of cancer risk in all these unexplained families makes it difficult to carry out an individualized genetic counseling.

Next-generation sequencing (NGS) has revolutionized cancer genomics research, and can be used to search for Mendelian disease genes in an unbiased manner by sequencing the entire protein-coding sequence of known predisposition genes [14]. The practice of genetic testing is rapidly evolving owing to the recent introduction of multigene panels for the diagnosis of hereditary cancer [15]. Multigene panels can be a cost and time-effective alternative to sequentially testing multiple genes. Virtually all multigene panels include high-penetrance genes that establish the risk of a particular type of cancer (such as breast or colon), but also many moderate and low-risk genes. This challenges the personalized management of guidelines when a pathogenic mutation is found, since the phenotypic spectrum and penetrance are less defined or unknown for the latter [16]. The TruSight Cancer Sequencing Panel has been developed by Illumina in collaboration with experts in cancer genomics, and targets a set of 94 well-known cancer-predisposing genes.

The purpose of the present study is the use of multigene panel testing for the diagnosis of hereditary cancer in individuals from high-risk colorectal cancer families.

## Methods

### Patient selection

A total of 1204 high-risk CRC families have been referred for genetic counseling and/or gene testing for Lynch Syndrome at the Cancer Genetic Clinic of Hospital Clinico San Carlos between the years 2000 and 2016. Among them, 393 families fulfilled the Amsterdam I/II or Bethesda clinical criteria and were molecularly characterized by the study of MSI and/or MMR protein expression in the tumor, and the screening of germline MMR gene mutations (*MLH1*, *MSH2*, *MSH6*, *PMS2* and *EPCAM*, located upstream of *MSH2*) by HRM followed by Sanger sequencing [17,18]. Only 141 families in which a pathogenic germline MMR mutation was found were diagnosed with Lynch Syndrome, while the rest of the families could not be explained by these single-gene analyses. Other genes such as *POLE*, *POLD1* and *NTHL1* were also studied with no positive outcome.

**Fig 1:**
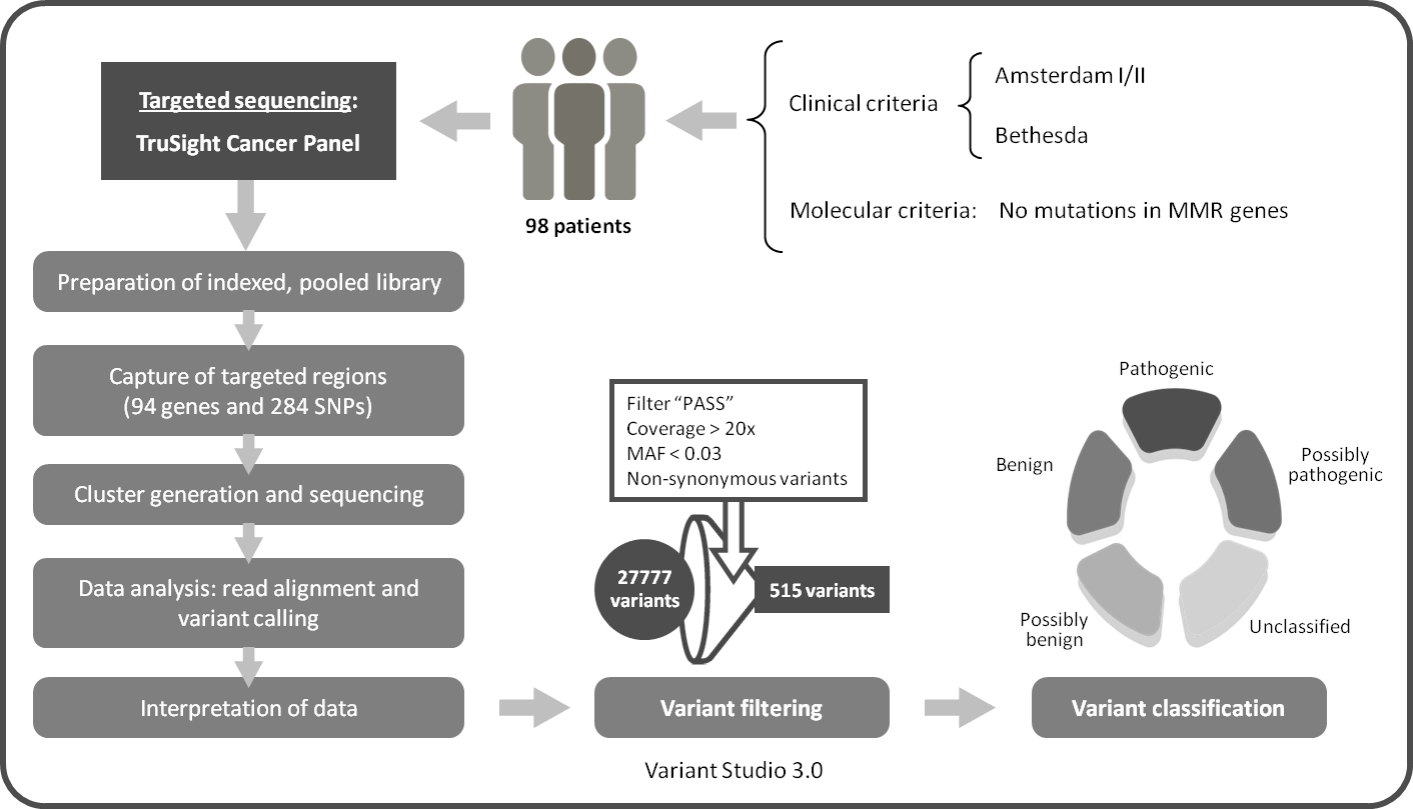
Next generation sequencing workflow using the TruSight cancer sequencing panel.

For the present study, we selected 98 patients from those unexplained high-risk CRC families for the test of a multigene cancer panel by NGS (Fig 1). The prioritization of the families was based on the absence of MMR proteins, presence of MSI, lower age at diagnosis or higher number of cancer patients in the pedigree. An *MLH1* mutation carrier was added as a positive control. Participants were asked to donate 10ml of blood at the time of their initial visit. Personal and family histories were obtained from the proband and participating relatives, and cancer diagnoses were confirmed by medical and pathology records. A written informed consent was signed by each participant, and the study was approved wirh an internal code n° 16/204-E_BS by the Clinical Investigation Ethics Committee (CEIC) from the Hospital Clinico San Carlos.

### Genomic DNA extraction

Peripheral-blood genomic DNA was extracted with the MagnaPure Compact extractor (Roche, Switzerland) according to the manufacturer’s recommended protocol. DNA concentration was measured by Nanodrop (Life Technologies, USA) and Qubit (Life Technologies, USA). The DNA integrity was evaluated by agarose gel electrophoresis.

### Next generation sequencing of multiplex PCR amplicons

The patients were tested with the TruSight Cancer Sequencing Panel, targeting 94 genes known to play a role in cancer predisposition (S1 Table). The kit includes more than 5,000 highly-optimized probes (80 mer) that cover the genes and that have been constructed against the human NCBI37/hg19 reference genome. The integrated sample preparation and sequencing was done following the protocol from Illumina, using the Nextera enrichment method and as little as 50ng of DNA for the library. The NGS workflow is summarized in Fig 1.

### Data analysis

Sequencing data was analyzed by the MySeq Reporter Software. After the demultiplexing and FASQ file generation, the reads were aligned against the *Homo sapiens* reference genome hg19 to create the BAM files. The genome analysis software Toolkit was used to perform the variant calling and generate the VCF files. After this, Variant Studio 3.0 was used for the variant filtering and annotation. Only variants with 95% of exon covered, labeled as “PASS”, with a minimum coverage of 20X and a minor allele frequency (MAF) <0.03 were selected. The variants present in more than 10 different patients were discarded. Regarding the consequence, we only considered missense, nonsense, splicing, in frame and frameshift variants.

### Copy number variation analysis

In order to look for DNA copy number variations (CNVs), the BAM files obtained by NGS were analyzed using the Enrichment v3.0.0 app from Illumina’s BaseSpace. Those CNVs observed with a quality value greater than 4 and a length greater than 10kb were labeled as “PASS” and selected for further evaluation. Variants present in 4 or more different patients were discarded. The areas affected by potential variations were then examined by the Database of Genomic Variants (DGV) [19] and the Integrative Genomics Viewer (IGV) [20]. CNVs with a frequency above 1% and described by at least two studies were also eliminated. Multiplex ligation-dependent probe amplification (MLPA) was finally carried out in samples 786 and 2954 to confirm *BRCA2*, *MSH2* and *EPCAM* structural variations using SALSA MLPA probe mixes P045, P003 and P072-B2 (MRC-Holland, Netherlands), according to the manufacturer’s protocol.

### Confirmation of the variants

All clinically actionable variants identified by NGS were validated by Sanger sequencing on a 3130 Genetic Analyzer, with the BigDye Terminator v3.1 Cycle Sequencing Kit (Thermo Fisher Scientific, USA). Sequencing data was aligned against the appropriate reference sequences and analyzed using the Sequencing Analysis Software v5.3.1 (Gene Codes Corp., USA). Unconfirmed variants were eliminated from the results.

### Annotation and variant classification

Variants were annotated according to nomenclature recommendations from the Human Genome Variation Society (www.hgvs.org/mutnomen) and further categorized according to the American College of Medical Genetics and Genomics [21] as: benign (class 1), likely benign (class 2), uncertain significance (class 3), likely pathogenic (class 4) and pathogenic (class 5). The following public databases were used for the interpretation of the variants: ClinVar (https://www.ncbi.nlm.nih.gov/clinvar/), UMD (http://www.umd.be/), InSight (https://www.insight-group.org/variants/databases/), COSMIC (https://cancer.sanger.ac.uk/cosmic) and Ensembl (http://www.ensembl.org/). Last access: April 2018. Four different *in silico* programs were used for the damage prediction of missense variants (Sift, Provean, PolyPhen-2 and MutationTaster).

### Data accesibility

Sequencing data have been deposited at the NCBI SRA archive with BioProject record PRJNA474807 and SRA accession SUB4117212.

## Results

### Patient samples

Germline DNA was analyzed from 98 patients belonging to high-risk CRC families using the Illumina TruSight Cancer Sequencing Panel, which targets a set of 94 genes known to play a role in cancer predisposition. All patients had been previously tested for Lynch Syndrome by IHC/MSI analyses in the tumor and/or germline MMR single-gene mutation screening, with a negative result for the latter. Characteristics of the studied cohort are detailed in Table 1. The studied families were predominantly affected by CRC; however, other malignancies were observed in some family members, including endometrial, gastric, ovarian, breast, renal and pancreatic cancers. The mean age at diagnosis was 49.1 years old. Most of the carcinomas had an MSS phenotype and presence of the MMR proteins in the tumor. Only 11 patients showed an MSI phenotype, 7 of which had absence of at least one MMR protein (but with no germline MMR mutations detected). The majority of CRCs were Dukes B or C, and nearly all were left-sided.

**Table 1:** Clinical and molecular characteristics of the 98 probands.

	Amsterdam I (N = 31)		Amsterdam II (N = 22)		Bethesda (N = 45)		Total n° (N = 98)
**Gender**							
M	14		12		20		46
F	17		10		25		52
**Diagnosis age**							
<50	19		6		31		56
>50	12		16		14		42
**Tumor type**							
CRC	31		18		42		91
Breast	0		0		1		1
Gastric	0		1		1		2
Other tumors	0		3		1		4
**MSI result**							
MSI	6		1		4		11
MSS	19		17		27		63
Unknown	6		4		14		24
**IHC result**							
MMR-presence	21		16		18		55
MMR-absence	4		1		4		9
Unknown	6		5		23		34
**MMR gene test**							
MMR wt	28		21		33		82
Unknown	3		1		12		16

N: number of patients; M: male; F: female; CRC: colorectal cancer; MSI: microsatellite instability; MSS: microsatellite stable; IHC: immunohistochemistry; MMR: mismatch repair; wt: wild type.

### Results from the NGS targeted sequencing

Among the 98 patients, we found 19 pathogenic or likely pathogenic variants in 18 patients (18.4%), all of which were validated by Sanger sequencing. Table 2 shows the clinical and molecular features of the patients with these mutations. Out of the 18 patients, 8 had *MLH1*/*MSH6*/*PMS2* mutations and 10 carried non-MMR mutations. Four out of the 8 patients with MMR mutations had MSI tumors and absence of the corresponding MMR proteins, while another 3 patients had a discordant tumor screening: one (ID 820) with a frameshift mutation in *PMS2*, c.714_720dup (MSI/presence of PMS2), another (ID 7400) with a splicing mutation in *MLH1*, c.1731+4A>G (MSS/absence of MLH1/PMS2), and the last (ID 7934) with a missense mutation in *MLH1*, c.677G>T (MSI/presence of MLH1/PMS2). Patient ID 555 carried two MMR variants, one in *MLH1* (c.2050T>G, p.Y684D) and another in *PMS2* (c.825A>T, p.Q275H). This patient was diagnosed of CRC at 35 years old, belonged to an Amsterdam I family and had an MSI tumor with absence of MLH1/PMS2. *In silico* studies of both mutations showed that the *PMS2* mutation was neutral while the *MLH1* mutation was predicted to be highly damaging. On the other hand, all of the 10 families with pathogenic variants in non-MMR genes showed MSS tumors, and in one of the families (patient ID 1041) we found two pathogenic variants in *MUTYH* (Table 2). From the remaining patients, 55 (56.1%) were revealed to only carry variants of unknown significance (VUS) in 38 different genes (S2 Table), while 25 (25.5%) just carried polymorphisms.

**Table 2:** MMR status in tumors from patients with selected variants identified by the TruSight cancer sequencing panel.

Patient ID	Family Criteria	Cancer Type	Dx Age	MMR status (*MLH1* / *MSH2* / *MSH6* / *PMS2*)			Mutations detected by TruSight Cancer Panel	
				MSI	IHC	HRM	Gene	Variant
499	BETH	CRC	63	MSS	Presence	Wild Type	*BRIP1*	c.903del (p.L301FfsTer2)
555	AMS I	CRC	35	MSI-H	Absence MLH1/PMS2	Wild Type	*MLH1*	c.2050T>G (p.Y684D)
763	AMS I	CRC	47	MSS	Presence	Wild Type	*CHEK2*	c.349A>G (p.R117G)
820	AMS I	CRC	44	MSI-H	Presence	Wild Type	*PMS2*	c.714_720dup (p.F242HfsTer9)
987	AMS I	CRC	62	MSS	Presence	Wild Type	*SMAD4*	c.1194G>A (p.W398Ter)
1041	BETH	CRC	60	MSS	Presence	Wild Type	*MUTYH*	c.1187G>A (p.G396D) c.1227_1228dupGG (p.E410GfsTer43)
1144	AMS I	CRC	45	MSI-H	Absence MLH1/PMS2	Wild Type	*MLH1*	c.2141G>A (p.W714Ter)
1564	AMS II	CRC	58	MSS	Presence	Wild Type	*HNF1A*	c.92G>A (p.G31D)
1652	BETH	CRC	62	MSS	Presence	Wild Type	*XPC*	c.1001C>A (p.P334H)
1756	BETH	CRC	31	MSI-H	Absence MLH1/PMS2	Wild Type	*MLH1*	c.1896+2T>C
1803	AMS II	CRC	79	MSS	Presence	Wild Type	*MUTYH*	c.536A>G (p.Y179C)
1936	AMS I	CRC	47	MSI-H	Absence MSH2/MSH6	Wild Type	*MSH6*	c.1635_1636delAG (p.E546GfsTer16)
2291	AMS I	CRC	51	ND	ND	Wild Type	*PMS2*	c.903G>T (p.K301N)
2456	AMS II	Ovary	35	MSS	Presence	Wild Type	*TP53*	c.783-1G>A
2910	BETH	CRC	39	MSS	Presence	Wild Type	*APC*	c.3199C>T (p.Q1067Ter)
3775	AMS I	CRC	47	MSS	Presence	Wild Type	*MUTYH*	c.1187G>A (p.G396D)
7400	AMS II	CRC	39	MSS	Absence MLH1/PMS2	Wild Type	*MLH1*	c.1731+4A>G
7934	BETH	CRC	35	MSI-H	Presence	ND	*MLH1*	c.677G>T (p.R226L)

BETH: Bethesda; AMS I/II: Amsterdam I and II; CRC: colorectal cancer; Dx Age: age at diagnosis; MMR: mismatch repair; MSS: microsatellite stable; MSI-H: microsatellite instablility-high; IHC: immunohistochemistry; HRM: high resolution melting (for germline screening); ND: not determined.

It is worth noting that, in total, only three splicing variants were not validated by Sanger sequencing (*EZH2* c.1947+1G>T, *MSH2* c.942+2T>G and *MLH1* c.1059-1G>A) and were eliminated from the data. The CNVs were also analyzed as described in Materials and Methods (S3 Table). However, potential structural variations were discarded by the study of SNPs in the corresponding chromosome localization using IGV. It was not possible to analyze some CNVs due to the low coverage or absence of SNPs in the region. Three CNVs affecting *BRCA2*, *MSH2* and *EPCAM* were not confirmed in 2 of the samples (786 and 2954) by MLPA.

### Type, prediction and frequency of likely pathogenic mutations by gene

Out of the 19 germline pathogenic or likely pathogenic mutations detected, 8 (42.1%) were found in MMR genes (5 in *MLH1*, 1 in *MSH6* and 2 in *PMS2*). The remaining 11 mutations were detected in other cancer predisposing genes, including *BRIP1* (n = 1), *CHEK2* (n = 1), *SMAD4* (n = 1), *MUTYH* (n = 4), *HNF1A* (n = 1), *XPC* (n = 1), *TP53* (n = 1), and *APC* (n = 1). The type, prediction and frequency of all the mutations can be observed in Table 3. Among them, there were 9 missense, 4 frameshift, 3 stop-gained and 3 splice site variants. All these variants were rare, and 11 had frequency data not available (NA) in ExAc nor in GnomAD (Table 3). 15 of these mutations were classified as pathogenic or likely pathogenic by the ClinVar and/or InSight databases, following the 5-tier classification system proposed by Plon and colleagues [22]. In addition, 4 of them did not appear in any of the variant databases mentioned earlier, but were considered likely pathogenic due to the type of mutation, *in silico* predictions and/or the molecular features of the tumor. These novel variants were: *SMAD4* c.1194G>A, *PMS2* c.714_720dup, *MSH6* c.1635_1636del and *MLH1* c.2050T>G.

**Table 3:** Pathogenic and likely pathogenic variants by gene identified by the TruSight cancer sequencing panel.

Patient ID	Family Criteria	Gene	Variant (c.)	Variant (p.)	Type of Mutation	Prediction	Database	Frequency (ExAC)
499	BETH	*BRIP1*	c.903delG	p.Leu301PhefsTer2	Frameshift	Pathogenic	ClinVar	NA
555	AMS I	*MLH1*	c.2050T>G	p.Tyr684Asp	Missense	Likely Pathogenic^#^	Novel	NA
763	AMS I	*CHEK2*	c.349A>G	p.Arg117Gly	Missense	Likely Pathogenic	ClinVar	0.00013
820	AMS I	*PMS2*	c.714_720dup	p.Phe242HisfsTer9	Frameshift	Pathogenic	Novel	NA
987	AMS I	*SMAD4*	c.1194G>A	p.Trp398Ter	Stop gained	Pathogenic	Novel^†^	NA
1041	BETH	*MUTYH*	c.1187G>A c.1227_1228dup	p.Gly396Asp p.Glu410GlyfsTer43	Miss, SP region Frameshift	Pathogenic* Pathogenic*	ClinVar, InSiGHT ClinVar, InSiGHT	0.00280
								0.00015^¶^
1144	AMS I	*MLH1*	c.2141G>A	p.Trp714Ter	Stop gained	Pathogenic	ClinVar, InSiGHT	NA
1564	AMS II	*HNF1A*	c.92G>A	p.Gly31Asp	Missense	Likely pathogenic	ClinVar	0.00071
1652	BETH	*XPC*	c.1001C>A	p.Pro334His	Missense	Likely pathogenic	ClinVar	0.00286
1756	BETH	*MLH1*	c.1896+2T>C	-	Splice donor	Likely pathogenic	ClinVar, InSiGHT	NA
1803	AMS II	*MUTYH*	c.536A>G	p.Tyr179Cys	Missense	Pathogenic*	ClinVar, InSiGHT	0.00162
1936	AMS I	*MSH6*	c.1635_1636del	p.Glu546GlyfsTer16	Frameshift	Pathogenic	Novel	NA
2291	AMS I	*PMS2*	c.903G>T	p.Lys301Asn	Miss, SP region	Likely Pathogenic	ClinVar, InSiGHT	0.000008
2456	AMS II	*TP53*	c.783-1G>A	-	Splice acceptor	Pathogenic	ClinVar, IARC *TP53*	NA
2910	BETH	*APC*	c.3199C>T	p.Gln1067Ter	Stop gained	Pathogenic	ClinVar, InSiGHT	NA
3775	AMS I	*MUTYH*	c.1187G>A	p.Gly396Asp	Miss, SP region	Pathogenic*	ClinVar, InSiGHT	0.00280
7400	AMS II	*MLH1*	c.1731+4A>G	-	Splice donor	Likely Pathogenic	ClinVar, InSiGHT	NA
7934	BETH	*MLH1*	c.677G>T	p. Arg226Leu	Miss, SP region	Likely Pathogenic	ClinVar, InSiGHT	NA

BETH: Bethesda; AMS I/II: Amsterdam I and II; Miss: missense; SP: splicing; NA: not available

^#^predicted to be probably damaging by *in silico* tools

*only causal in homozygosis or in co-occurrence with other mutations

^†^described in COSMIC

^¶^frequency data from gnomAD exomes.

### Segregation studies

Unfortunately, segregation studies could not be performed in most of the families, given the unavailability of other family members. Among the few families in which the segregation was studied is the one with the *MLH1* c.2050T>G mutation (ID 555). However, this was not very informative, since all the affected members were deceased and only healthy relatives could be tested. Out of the 4 members analyzed, only one carried the variant but was too young to have developed the disease. On the other hand, in the family of ID 1144 (*MLH1* c.2141G>A) 9 relatives were studied, one of whom had developed CRC at the age of 56. As expected, this affected member was shown to carry the mutation, together with another 5 healthy members who were all under 60 (3 of them especially young) and will follow the surveillance recommendations of the Genetic Counseling Unit. For the *CHEK2* variant (c.349A>G), only one distant relative with polyps could be tested and was wild type for the mutation. However, this member also had a CRC history coming from the other side of the family, so no conclusions can be drawn from this result. Finally, the son of participant ID 3775 (monoallelic *MUTYH* c.1187G>A mutation) was also evaluated with a negative result. Although this member had also been reported to have some polyps, not much information was available.

### Frequency of VUS in cancer susceptibility genes

**Fig 2:**
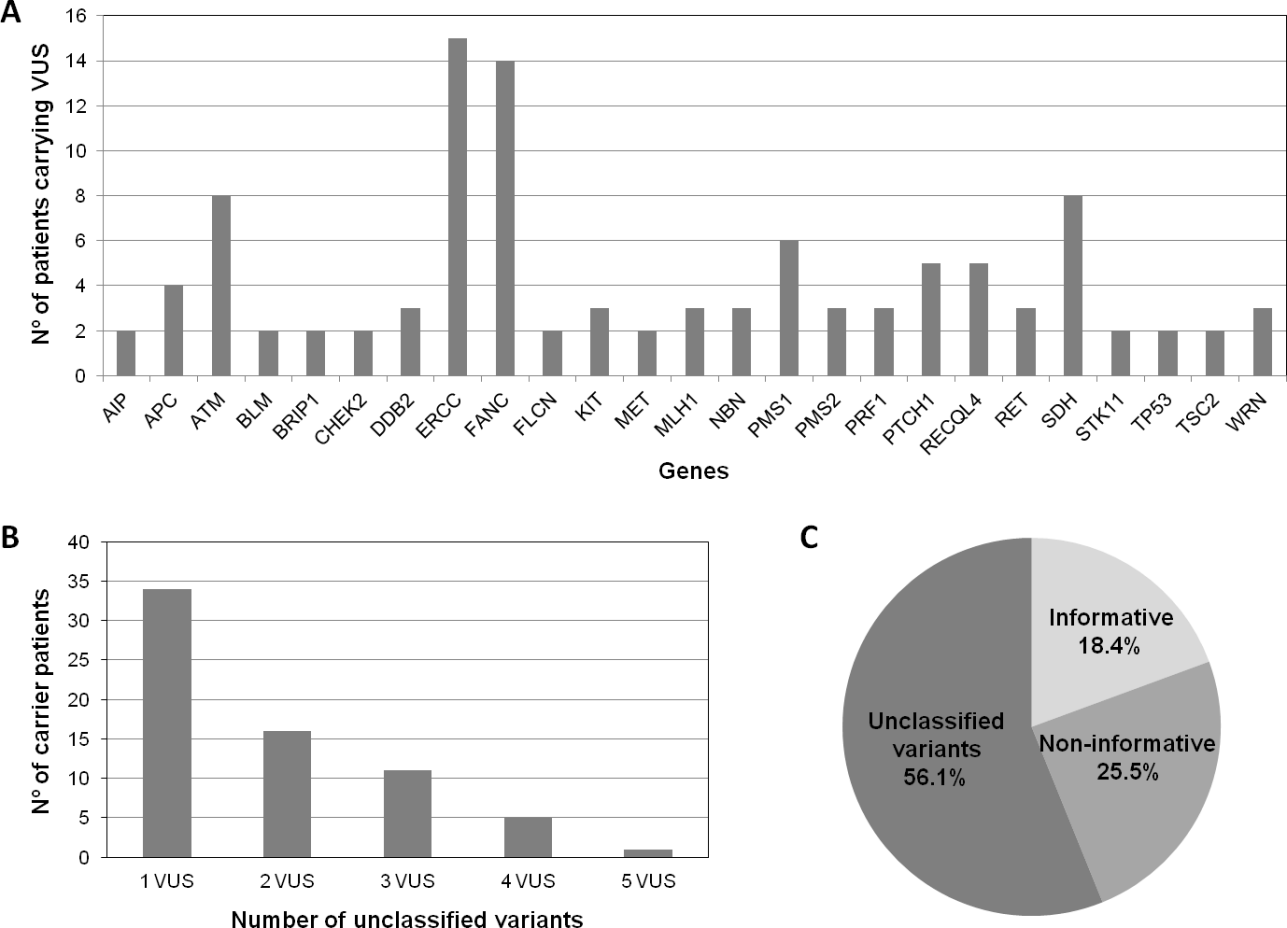
Class 3 variants found in genes implicated in hereditary cancer and clinical experience. A) Number of rare VUS (MAF<0.005) per gene or group of genes, in those cancer susceptibility genes with at least 2 filtered VUS. The different *FANC*, *ERCC* and *SDH* genes were grouped together for simplification. B) Number of unclassified variants per patient of the study cohort. C) Clinical practice experience with multigene panel study. VUS: variants of unknown significance.

All the variants found by NGS were analyzed by ClinVar, and those variants classified as class 3 were selected as VUS. Only those VUS with a very low frequency (<0.005) in ExAc are included in S2 Table. The VUS in cancer genes can be grouped by their functional effect; Fig 2A shows that most of the VUS selected were located in genes involved in DNA repair mechanisms, tumor suppressor genes and proto-oncogenes. Fig 2B shows that the relationship between the number of patients and the number of variants per patient is inversely proportional: 33 patients had 1 VUS, while only one patient (ID 1008) had 5 VUS, and was curiously a patient with a strong family history. Eleven patients (IDs 499, 763, 820, 987, 1041, 1144, 1564, 1652, 1936, 3775 and 7934) with VUS also had a concomitant deleterious mutation (data shown in Tables 3 and S2 Table). Among the 55 patients that carried only VUS (56.1%), 31 fulfilled the Amsterdam I/II clinical criteria.

## Discussion

Approximately half of HNPCC families carry a germline, pathogenic mutation in one of the MMR genes and are thus considered Lynch Syndrome families. The other half does not present any evidence of MMR deficiency and the genetic basis underlying their cancer predisposition remains unknown, reason for which they are called FCCTX [13,23,24]. However, unexplained families also comprise cases which present MMR defects and/or MSI in the tumors but lack the corresponding germline MMR mutations. All together, these families represent a significant number of cases at the Genetic Counseling Units and they are considered a problem in the clinic. Despite this, the genetic understanding of hereditary CRC syndromes has grown over the years, leading to an increasing request for genetic testing [25]. The limitation of the old screening methods due to their lower sensitivity and the reduced number of genes studied has led to the rise of multigene panel testing in oncology [16,26], and given the advantages of analyzing multiple genes, the benefits of its application in the clinical practice are obvious [15,27].

In this study, 98 unexplained families were reanalyzed using Illumina's NGS TruSight Cancer Sequencing Panel, which targets 94 genes known to play a role in cancer predisposition. The panel testing identified 8 MMR mutations in our cohort (5 in *MLH1*, 1 in *MSH6* and 2 in *PMS2*), 7 of which were found in patients whose tumors showed an altered MMR status (MSI and/or absence of MMR). These cases were missed by our prior screening, and are thus a result from improved testing for these genes. Although these patients represent most of the Lynch-suspected families included in our study, there were another 6 families that were not resolved. The panel also allowed the identification of mutations in other well-known CRC high-penetrance genes, such as *MUTYH* (biallelic), *APC*, *SMAD4* and *TP53*, as well as in moderate-penetrance genes like *MUTYH* (monoallelic), *CHEK2*, *HNF1A*, *BRIP1* and *XPC*. In total, pathogenic or likely pathogenic mutations were found in 18.4% of our cohort, while high-penetrance mutations represented 12.2% of the studied patients.

Four of the identified variants were novel and had not been previously described in any of the variant databases checked (ClinVar, InSiGHT and UMD), although *SMAD4* c.1194G>A had been reported at somatic level in COSMIC. There is enough evidence to claim that the 3 deleterious novel variants (*SMAD4* c.1194G>A, *PMS2* c.714_720dup and *MSH6* c.1635_1636del) are pathogenic, so they will be added to the public databases for future reference. Regarding the missense variant (*MLH1* c.2050T>G), it was present in a patient whose tumor was MSI with absence of MLH1/PMS2. In addition, *in silico* studies showed the change as likely pathogenic. However, the segregation studies performed were not very informative given the lack of affected living relatives, so more studies are needed in order to confirm the pathogenicity of this variant.

Among all the non-MMR genes, *APC*, *MUTYH* and *SMAD4* are well known to be implicated in CRC, specifically in polyposis. It is worth noting that the patient in which the *APC* mutation was found had over 30 polyps. However, *APC* as well as other polyposis-associated genes had already been screened with no positive results (data not shown). For that reason, the family was added to our study on the grounds that it fulfilled the Bethesda criteria. Like the MMR mutations identified, this variant represents a false-negative of previous screenings. Regarding the biallelic *MUTYH* carrier, there was no information of the presence of polyps at the time of patient selection, but a deeper look into the family history revealed that the patient did present multiple polyps. On the contrary, neither the *SMAD4* nor the 2 monoallelic *MUTYH* mutation carriers showed any sign of polyposis to our knowledge. Nevertheless, the risk that monoallelic *MUTYH* mutations confer is uncertain, so it is unlikely that they are the only cause of cancer in the corresponding families [27].

The family that carried the *TP53* mutation was not a classic Li-Fraumeni family, but did fulfill the revised Chompret criteria. It had been classified as an Amsterdam II family because there was one member affected with ovary cancer and another 3 with CRC at early ages, but there were also 2 lung cancers within the family and a cousin of the proband had developed a sarcoma very young.

The remaining genes with pathogenic or likely pathogenic mutations had moderate or less defined cancer risks. In the first place, the *BRIP1* gene encodes a member of the RecQ DEAH helicase family that interacts with the BRCT repeats of BRCA1. The bound complex is important in the normal double-strand DNA break repair and appears to be involved in breast and ovarian cancer, where it acts as a tumor suppressor [28]. Rafnar et al. showed that women with *BRIP1* mutations have an increased risk for ovarian cancer that may be as much as 5 times higher than the risk in non-carriers [29]. Noteworthy, one of the *BRIP1* mutations reported by Rafnar et al. (c.1702_1703delAA, p.N568WfsTer9) was also found in a Spanish CRC patient [30], and the tumor showed a loss of the wild type allele in both studies. Here we have identified a different frameshift *BRIP1* mutation (c.903delG, p.L301FfsTer2) in a patient diagnosed with CRC at 63 years old. The family fulfilled the Bethesda criteria but was not very informative due to its reduced size and limited information. Therefore, we still cannot determine the risk for CRC that *BRIP1* mutations confer.

Another gene was *CHEK2*, a tumor suppressor that is activated when DNA is damaged or when DNA strands break. The c.1100delC mutation of *CHEK2* has been confirmed to confer an increased risk of breast cancer in women unselected by family history [31,32,33]. The lifetime risk of developing breast cancer among women with a *CHEK2* mutation has been reported to be approximately 25% [34]. In our cohort, we have found *CHEK2* c.349A>G (p.Arg117Gly), which was considered likely pathogenic by Shoda et al. and proved to produce a nonfunctional protein both by biochemical and bioinformatics analyses [35]. In addition, their results suggest that both of these mutations cannot act in a dominant-negative manner and that tumorigenesis associated with this mutation may be due to haploinsufficiency [35].

We also identified a mutation in *XPC*, a key component of the XPC complex that plays an important role in the early steps of global genome nucleotide excision repair and is involved in damage sensing and DNA binding. The *XPC* mutation found in our study (c.1001C>A, p.Pro334His) had been described as pathogenic in ClinVar by Johns Hopkins University. However, we have identified this mutation for the first time in a CRC patient, who was diagnosed at the age of 50 and did not have any cancer family history. The patient died soon after the diagnosis, not being able to get any additional information. Last but not least, HNF1A is a transcription factor that regulates tissue specific expression of many genes. This gene is implicated in diabetes and had been described in renal cancer, but its role in CRC is unknown. We are also describing the mutation *HNF1A* c.92G>A (p.Gly31Asp) in CRC for the first time.

An increasing number of studies have been published over the past few years addressing the benefits of NGS panel testing for the diagnosis of hereditary cancers as compared with the traditional targeted single-gene screenings. While all of them agree on the huge advantages of panel testing, especially due to its time and cost-efficiency and its higher sensitivity, the only question that remains open to debate is the selection of genes that should be included [36–40]. Here we have used a 94-gene panel, which is to our knowledge the highest number of genes reported in this kind of studies by far. However, this choice was made based on a number of reasons. On the one hand, we have learned from the Genetic Counseling Unit that there is a relatively frequent overlapping of phenotypes among the different hereditary cancer syndromes [36,37], since the tumor spectrum is sometimes wider than expected [27,41] and the information provided by the family is sometimes incomplete. Therefore, we believe that the best strategy is to group all the syndromes into one single cancer-predisposing multigene panel, as proposed by some other groups [27,36], instead of dividing patients based on their phenotypes.

On the other hand, we strongly support the incorporation of lesser-known genes to NGS panels on a research basis, since the additional cost of adding these genes is minimal [42], and with the intensity of current research the uncertainty of many emerging genes is likely to be resolved soon [38]. This means that some variants that are not informative at the moment may be actionable in the future [38]. In addition, by adding these genes to our panel we are contributing to the accumulation of international research data, which is the only way to continue improving our understanding of CRC genetics [39]. For this reason, we also believe it is highly valuable to include a detailed list of VUS (S2 Table), something that most published studies fail to do [38]. The number of VUS identified cannot be compared with other studies, though, since it is associated with the number of genes on the panel [40]. Despite the high number of genes included in the panel used for the present study, there were some genes that were left out, such as *POLE*, *POLD1*, *NTHL1* or *MSH3*. Although some of them (*POLE*, *POLD1* and *NTHL1*) had already been screened in our cohort, this is a limitation of our study. It goes without saying that we would definitely recommend that future panels used in clinical studies for colorectal cancer families should include those genes as well, for the same reasons that were discussed above.

The clinical practice experience obtained with this multigene panel is shown in Fig 2C. Among all the families that were screened only 18.4% were informative, although this group is underrepresented considering that only unexplained families from previous screenings were included in the study. Out of these, 66.7% carried likely pathogenic mutations in high-penetrance genes and could benefit from a true genetic counseling, taking measures such as reducing the surveillance in non-carriers, who would avoid the stress attached to the lack of awareness. Regarding moderate-penetrance genes, a study with a larger number of patients is needed in order to establish the exact risk they confer. The introduction of NGS panels in the clinical routine of the hospital will help us with this task. Those patients who were just informed of VUS (56.1%) would also take advantage of this measure, since the only thing we can do for now is to keep track of public databases, study the segregation and do functional studies when recommended in order to improve their genetic counseling in the future. The remaining patients (25.5%) were informed that no gene had been found to be involved in their cancer predisposition. Although data regarding lesser-known genes and VUS is highly valuable from a research point of view, participants should be always informed about the limited clinical actionability of testing for genes that are not associated with their phenotype or have moderate penetrance.

In conclusion, the detection of new pathogenic mutations in high-penetrance genes can contribute to the explanation of the cancer heritability in our families, changing the individual clinical management. The NGS panel approach has the advantage of analyzing multiple genes in multiple samples simultaneously, reducing costs and time and increasing the sensitivity in comparison to targeted single-gene screenings. Therefore, multigene panels should be included in clinical laboratories for the screening of all high-risk cancer families regardless of other analyses in the tumor. The number of genes to be included in these panels is debatable, though, and should fit the purposes of each study.

## Supporting information

S1 Table List of genes included in the TruSight cancer sequencing panel. (DOCX)

S2 Table Variants of unknown significance identified by the TruSight cancer sequencing panel (Illumina). (DOCX)

S3 Table Results from the CNVs analysis. (XLSX)
