# Supplementary material for: Novel genetic mutations detected by multigene panel are associated with hereditary colorectal cancer predisposition
Source: PLoS One. 2018 Sep 26;13(9):e0203885. doi: 10.1371/journal.pone.0203885 (PMC6157886; doi:10.1371/journal.pone.0203885)
Supplement: S1 Table — (DOCX) [file pone.0203885.s001.docx]

**Supplementary Table 1. List of genes included in the TruSight Cancer Sequencing Panel.**

| **Genes** | | | | | | | |
| --- | --- | --- | --- | --- | --- | --- | --- |
| *AIP* | *CDKN1C* | *CDH1* | *FANCE* | *KIT* | *PALB2* | *RET* | *SUFU* |
| *ALK* | *CDKN2A* | *CDK4* | *FANCF* | *MAX* | *PHOX2B* | *RHBDF2* | *TMEM127* |
| *APC* | *CEBPA* | *ERCC3* | *FANCG* | *MEN1* | *PMS1* | *RUNX1* | *TP53* |
| *ATM* | *CEP57* | *ERCC4* | *FANCI* | *MET* | *PMS2* | *SBDS* | *TSC1* |
| *BAP1* | *CHEK2* | *ERCC5* | *FANCL* | *MLH1* | *PRF1* | *SDHAF2* | *TSC2* |
| *BLM* | *CYLD* | *EXT1* | *FANCM* | *MSH2* | *PRKAR1A* | *SDHB* | *VHL* |
| *BMPR1A* | *DDB2* | *EXT2* | *FH* | *MSH6* | *PTCH1* | *SDHC* | *WRN* |
| *BRCA1* | *DICER1* | *EZH2* | *FLCN* | *MUTYH* | *PTEN* | *SDHD* | *WT1* |
| *BRCA2* | *DIS3L2* | *FANCA* | *GATA2* | *NBN* | *RAD51C* | *SLX4* | *XPA* |
| *BRIP1* | *EGFR* | *FANCB* | *GPC3* | *NF1* | *RAD51D* | *SMAD4* | *XPC* |
| *BUB1B* | *EPCAM* | *FANCC* | *HNF1A* | *NF2* | *RB1* | *SMARCB1* |  |
| *CDC73* | *ERCC2* | *FANCD2* | *HRAS* | *NSD1* | *RECQL4* | *STK11* |  |
