# Supplementary material for: Novel genetic mutations detected by multigene panel are associated with hereditary colorectal cancer predisposition
Source: PLoS One. 2018 Sep 26;13(9):e0203885. doi: 10.1371/journal.pone.0203885 (PMC6157886; doi:10.1371/journal.pone.0203885)
Supplement: S2 Table — (DOCX) [file pone.0203885.s002.docx]

**Supplementary Table 2.** Variants of unknown significance identified by the TruSight Cancer Sequencing Panel (Illumina).

| **Patient** | **Criteria** | **Gene** | **Variant (c.)** | **Variant (p.)** | **Variant type** | **dbSNP** | **Frequency (ExAc)** |
| --- | --- | --- | --- | --- | --- | --- | --- |
| 242 | BETH | *MEN1* | c.1296G>A | p.Leu432= | Missense variant | rs138770431 | 0.001025 |
|  |  | *MUTYH* | c.1276C>T | p.Arg426Cys |  | rs150792276 | 0.0007694 |
| 261 | AMS II | *FANCD2* | c.2872G>A | p.Val958Met | Missense variant | - | 0.00014 |
|  |  | *KIT* | c.844G>A | p.Val282Ile |  | rs771961192 | 0.00002 |
|  |  | *SDHD* | c.34G>A | p.Gly12Ser |  | rs34677591 | 0.007268 |
| 263 | AMS II | *CHEK2* | c.668G>A | p.Arg223His | Missense variant | rs137853009 | 0.00003 |
|  |  | *CHEK2* | c.973C>G | p.His325Asp | Missense, splice region | - | - |
|  |  | *MLH1* | c.1421G>A | p.Arg474Gln | Missense variant | rs63751083 | 0.000008 |
|  |  | *PRF1* | c.755A>G | p.Glu253Gly |  | rs28933375 | 0.005175 |
| 308 | BETH | *ATM* | c.8560C>T | p.Arg2854Cys | Missense variant | rs201958469 | 0.0001815 |
| 334 | AMS II | *PTCH1* | c.1306G>A | p.Asp436Asn | Missense variant | rs142274954 | 0.0007248 |
| 356 | BETH | *MET* | c.607T>A | p.Ser203Thr | Missense variant | rs200861145 | 0.0006819 |
|  |  | *PTCH1* | c.3388G>A | p.Ala1130Thr |  | rs766037357 | 0.00002 |
| 372 | BETH | *ERCC3* | c.2111C>T | p.Ser704Leu | Missense variant | rs4150521 | 0.002174 |
|  |  | *ERCC5* | c.1571C>T | p.Pro524Leu |  | rs201684551 | 0.0001484 |
|  |  | *ERCC5* | c.2636A>G | p.Asn879Ser |  | rs4150342 | 0.009517 |
|  |  | *RET* | c.2498G>A | p.Arg833His |  | rs587782636 | 0.00003 |
| 419 | BETH | *DDB2* | c.738G>A | p.Thr246= | Missense variant | rs144266685 | 0.002514 |
| 436 | AMS II | *ERCC4* | c.1727G>C | p.Arg576Thr | Missense variant | rs1800068 | 0.0005441 |
| 449 | AMS I | *SDHB* | c.487T>C | p.Ser163Pro | Missense variant | rs33927012 | 0.01254 |
| 456 | BETH | *FANCC* | c.28T>G | p.Cys10Gly | Missense variant | rs147479204 | 0.00001 |
| 496 | BETH | *NBN* | c.643C>T | p.Arg215Trp | Missense variant | rs34767364 | 0.002953 |
| 499 | BETH | *PRF1* | c.755A>G | p.Asn252Ser | Missense variant | rs28933375 | 0.005175 |
| 504 | AMS I | *PTCH1* | c.1306G>A | p.Asp436Asn | Missense variant | rs142274954 | 0.0007248 |
| 556 | BETH | *APC* | c.3386T>C | p.Leu1129Ser | Missense variant | rs143638171 | 0.001617 |
|  |  | *SDHB* | c.487T>C | p.Ser163Pro |  | rs33927012 | 0.01254 |
| 557 | AMS I | *BLM* | c.968A>G | p.Lys323Arg | Missense variant | rs146504061 | 0.0003046 |
| 581 | AMS I | *ERCC4* | c.1727G>C | p.Arg576Thr | Missense variant | rs1800068 | 0.0005441 |
|  |  | *FLCN* | c.1333G>A | p.Ala445Thr |  | rs41419545 | 0.002718 |
|  |  | *PTCH1* | c.2173C>T | p.Pro725Ser |  | rs149258400 | 0.0008566 |
| 594 | AMS I | *BRIP1* | c.1871C>T | p.Ser624Leu | Missense variant | rs587781321 | - |
|  |  | *KIT* | c.2554G>A | p.Val852Ile |  | rs555650901 | 0.00003 |
| 622 | AMS II | *PMS2* | c.2149G>A | p.Val717Met | Missense variant | rs201671325 | 0.0009054 |
|  |  | *RECQL4* | c.2636C>A | p.Pro879His |  | rs137975310 | 0.01114 |
| 670 | AMS I | *FANCM* | c.527C>T | p.Thr176Ile | Missense variant | rs77374493 | 0.004376 |
|  |  | *RECQL4* | c.1847A>G | p.Asn616Ser |  | rs199654783 | 0.0005285 |
| 675 | BETH | *NBN* | c.643C>T | p.Arg215Trp | Missense variant | rs34767364 | 0.002953 |
| 685 | BETH | *FANCA* | c.2574C>G | p.Ser858Arg | Missense variant | rs17233141 | 0.01031 |
|  |  | *FANCF* | c.557C>T | p.Ala186Val |  | rs113910234 | 0.009135 |
| 694 | BETH | *DDB2* | c.738G>A | p.Thr246= | Missense variant | rs144266685 | 0.002514 |
| 763 | AMS I | *ERCC4* | c.1727G>C | p.Arg576Thr | Missense variant | rs1800068 | 0.0005441 |
|  |  | *PMS1* | c.605G>A | p.Arg202Lys |  | rs2066459 | 0.01205 |
|  |  | *WRN* | c.3101A>T | p.Tyr1034Phe |  | rs200370409 | 0.0003624 |
| 820 | AMS I | *ATM* | c.749G>A | p.Arg250Gln | Missense variant | rs56123940 | 0.00004 |
|  |  | *SDHC* | c.490A>T | p.Met164Leu |  | rs200375156 | 0.0006113 |
| 885 | BETH | *BLM* | c.2333C>G | p.Ser778Cys | Missense variant | rs139610577 | 0.0001318 |
|  |  | *STK11* | c.1211C>T | p.Ser404Phe |  | rs200078204 | 0.0009281 |
| 923 | AMS II | *RHBDF2* | c.414G>T | p.Gln138His | Missense variant | rs763124357 | 0.00009 |
| 987 | AMS I | *ATM* | c.2289T>A | p.Phe763Leu | Missense variant | rs34231402 | 0.0005112 |
| 1008 | AMS I | *AIP* | c.896C>T | p.Ala299Val | Missense variant | rs148986773 | 0.0004275 |
|  |  | *APC* | c.6985A>G | p.Ile2329Val |  | rs146048493 | 0.0002822 |
|  |  | *ERCC5* | c.2636A>G | p.Asn879Ser |  | rs4150342 | 0.009517 |
|  |  | *PMS1* | c.1643A>G | p.Lys548Arg |  | rs61736576 | 0.001297 |
|  |  | *SDHD* | c.34G>A | p.Gly12Ser |  | rs34677591 | 0.007268 |
| 1041 | BETH | *CDH1* | c.125C>T | p.Pro42Leu | Missense variant | rs876659333 | - |
| 1144 | AMS I | *FANCM* | c.2859A>C | p.Lys953Asn | Missense variant | rs142864437 | 0.00112 |
|  |  | *PTEN* | c.235G>A | p.Ala79Thr |  | rs202004587 | 0.0001343 |
|  |  | *WRN* | c.3785C>G | p.Thr1262Arg |  | rs78488552 | 0.002738 |
| 1192 | AMS I | *APC* | c.3386T>C | p.Leu1129Ser | Missense variant | rs143638171 | 0.001617 |
| 1215 | AMS I | *RET* | c.1529C>T | p.Ala510Val | Missense variant | rs201745826 | 0.0003178 |
|  |  | *TP53* | c.329G>A | p.Arg110His |  | rs11540654 | 0.00004 |
|  |  | *WT1* | c.745C>T |  |  |  |  |
|  |  |  |  | p.Pro249Ser |  | rs2234584 | 0.0003817 |
| 1217 | AMS I | *RECQL4* | c.3062G>A | p.Arg1021Gln | Missense variant | rs34666647 | 0.00396 |
| 1391 | AMS I | *BMPR1A* | c.1327C>T | p.Arg443Cys | Missense variant | rs35619497 | 0.0006095 |
|  |  | *RAD51C* | c.492T>G | p.Phe164Leu |  | rs573992101 | 0.00003 |
| 1409 | BETH | *FANCA* | c.2574C>G | p.Ser858Arg | Missense variant | rs17233141 | 0.01031 |
| 1564 | AMS II | *ATM* | c.2572T>C | p.Phe858Leu | Missense variant | rs1800056 | 0.009149 |
|  |  | *MLH1* | c.1852A>G | p.Lys618Glu |  | rs35001569 | 0.003418 |
|  |  | *PMS1* | c.605G>A | p.Arg202Lys |  | rs2066459 | 0.01205 |
| 1652 | BETH | *PMS2* | c.2182A>G | p.Thr728Ala | Missense variant | rs141893001 | 0.005634 |
|  |  | *SDHD* | c.149A>G | p.His50Arg |  | rs11214077 | 0.006515 |
| 1769 | BETH | *DDB2* | c.738G>A | p.Thr246= | Missense variant | rs144266685 | 0.002514 |
|  |  | *PMS1* | c.605G>A | p.Arg202Lys |  | rs2066459 | 0.01205 |
|  |  | *RECQL4* | c.3062G>A | p.Arg1021Gln |  | rs34666647 | 0.00396 |
|  |  | *SLX4* | c.2924C>T | p.Pro975Leu |  | rs114472821 | 0.004021 |
| 1818 | BETH | *BRIP1* | c.2220G>T | p.Gln740His | Missense variant | rs45589637 | 0.0004614 |
|  |  | *FLCN* | c.552C>A | p.Asn184Lys |  | rs143525924 | 0.00003 |
|  |  | *SMAD4* | c.647A>G | p.Asn216Ser |  | rs138386557 | 0.000008 |
| 1819 | AMS I | *AIP* | c.896C>T | p.Ala299Val | Missense variant | rs148986773 | 0.0004275 |
| 1832 | BETH | *FH* | c.1431_1433dupAAA | p.Lys477dup | Inframe insertion | rs367543046 | 0.0009071 |
| 1876 | BETH | *EGFR* | c.2024G>A | p.Arg675Gln | Missense variant | rs150423237 | 0.0002151 |
|  |  | *ERCC4* | c.1135C>T | p.Pro379Ser |  | rs1799802 | 0.003769 |
|  |  | *PMS1* | c.1643A>G | p.Lys548Arg |  | rs61736576 | 0.001297 |
|  |  | *STK11* | c.617C>T | p.Ala206Val |  | rs764244639 | 0.00002 |
| 1936 | AMS I | *PMS2* | c.1211C>G | p.Pro404Arg | Missense variant | rs536111818 | 0.00005 |
| 2058 | AMS I | *TSC2* | c.5383C>T | p.Arg1795Cys | Missense variant | rs45517423 | 0.001221 |
| 2134 | BETH | *RET* | c.3149G>A | p.Arg1050Gln | Missense variant | rs200956659 | 0.00004 |
| 2157 | AMS II | *ERCC3* | c.2080G>A | p.Ala694Thr | Missense variant | rs151216904 | 0.0007413 |
|  |  | *MLH1* | c.1852A>G | p.Lys618Glu |  | rs35001569 | 0.003418 |
|  |  | *PMS1* | c.1609G>A | p.Glu537Lys |  | rs151325573 | 0.00189 |
| 2166 | AMS I | *FANCF* | c.373G>A | p.Asp125Asn | Missense variant | rs61752920 | 0.006258 |
|  |  | *RECQL4* | c.3317G>A | p.Arg1106His |  | rs34236392 | 0.0002619 |
| 2346 | AMS I | *FANCD2* | c.4396C>A | p.Gln26His | Missense variant | rs45510294 | 0.0005716 |
|  |  | *TSC2* | c.1754G>A | p.Arg585His |  | rs397515063 | 0.00002 |
| 2359 | BETH | *ERCC2* | c.2083C>T | p.Arg695Cys | Missense variant | rs201392911 | 0.0001238 |
|  |  | *NBN* | c.1999T>C | p.Ser667Pro |  | rs587780091 | 0.00005 |
| 2428 | AMS II | *APC* | c.3920T>A | p.Ile1307Lys | Missense variant | rs1801155 | 0.001693 |
|  |  | *ERCC5* | c.2636A>G | p.Asn879Ser |  | rs4150342 | 0.009517 |
| 2457 | AMS I | *FANCM* | c.5224A>G | p.Ile1742Val | Missense variant | rs143662421 | 0.008398 |
|  |  | *PTCH1* | c.1306G>A | p.Asp436Asn |  | rs142274954 | 0.0007248 |
| 2496 | AMS I | *FANCI* | c.1573A>G | p.Met525Val | Missense variant | rs144908351 | 0.002134 |
| 2642 | AMS II | *MET* | c.1451A>G | p.His484Arg | Missense variant | rs781545528 | 0.00005 |
|  |  | *SDHD* | c.149A>G | p.His50Arg |  | rs11214077 | 0.006515 |
|  |  | *WRN* | c.2735T>G | p.Ile912Ser | Missense, splice region | rs11574323 | 0.000514 |
| 2779 | BETH | *ATM* | c.4258C>T | p.Leu1420Phe | Missense variant | rs1800058 | 0.01271 |
| 3317 | BETH | *ATM* | c.1229T>C | p.Val410Ala | Missense variant | rs56128736 | 0.002166 |
|  |  | *FANCF* | c.373G>A | p.Asp125Asn |  | rs61752920 | 0.006258 |
|  |  | *MUTYH* | c.1276C>T | p.Arg426Cys |  | rs150792276 | 0.0007694 |
|  |  | *SUFU* | c.1105G>A | p.Val369Ile |  | rs149449923 | 0.00009 |
| 3613 | BETH | *PRF1* | c.755A>G | p.Asn252Ser | Missense variant | rs28933375 | 0.005175 |
| 3775 | AMS I | *ERCC4* | c.2117T>C | p.Ile706Thr | Missense variant | rs1800069 | 0.0014 |
| 4103 | AMS II | *ATM* | c.1595G>A | p.Cys532Tyr | Missense variant | rs35963548 | 0.0002072 |
| 4106 | BETH | *ERCC3* | c.2080G>A | p.Ala694Thr | Missense variant | rs151216904 | 0.0007413 |
|  |  | *ERCC5* | c.2636A>G | p.Asn879Ser |  | rs4150342 | 0.009517 |
|  |  | *TP53* | c.587G>A | p.Arg196Gln |  | rs483352697 | - |
| 4131 | AMS I | *DIS3L2* | c.1447C>G | p.Arg483Gly | Missense variant | rs186865544 | 0.0006803 |
|  |  | *MSH6* | c.2281A>G | p.Arg761Gly |  | rs199876321 | 0.00003 |
|  |  | *SDHD* | c.149A>G | p.His50Arg |  | rs11214077 | 0.006515 |
| 5074 | AMS II | *ATM* | c.1229T>C | p.Val410Ala | Missense variant | rs56128736 | 0.002166 |
| 6695 | BETH | *FANCA* | c.*647G>C | - | 3' UTR variant | rs17227403 | 0.003395 |
| 7180 | AMS II | *KIT* | c.821C>T | p.Thr274Met | Missense variant | rs138585275 | 0.0004618 |
| 7555 | BETH | *ERCC4* | c.1135C>T | p.Pro379Ser | Missense variant | rs1799802 | 0.003769 |
| 7934 | BETH | *FANCM* | c.527C>T | p.Thr176Ile | Missense variant | rs77374493 | 0.004376 |

AMS I/II: Amsterdam I and II; BETH: Bethesda
